# Pennsylvania Hospital—Surgical Wards

**Published:** 1851-09

**Authors:** Addinell Hewson

**Affiliations:** Resident Physician; Pennsylvania Hospital


### PENNSYLVANIA HOSPITAL—*Surgical Wards.—Service of* DR. FOX.

*Cases discharged since July 1st, 1851.*

| | Cured. | Relieved. | Died. | Total. |
|---|---|---|---|---|
| Abscess of thigh, - - - - - | 0 | 0 | 1* | 1 |
| Bite of dog, - - - - - | 1 | 0 | 0 | 1 |
| Burns, - - - - - | 0 | 0 | 2 | 2 |
| Concussion of spine, - - - - - | 1 | 1 | 0 | 2 |
| Contusions, - - - - - | 7 | 2 | 0 | 9 |
| Conjunctivitis, - - - - - | 3 | 1 | 0 | 4 |
| Corneitis, - - - - - | 0 | 1 | 0 | 1 |
| Disease of thumb, (scrofulous,) - - | 1† | 0 | 0 | 1 |
| " of knee, - - - - - | 1 | 1 | 0 | 2 |
| Dislocations 3, viz : | | | | |
| Shoulder, - - - - | 1 | 0 | 0 | 1 |
| Hip, - - - - - | 2 | 0 | 0 | 2 |
| Fistula in ano, - - - - - | 1 | 1 | 0 | 2 |
| Fractures, simple, 26, viz : | | | | |
| Inferior maxilla, - - - - - | 1 | 0 | 0 | 1 |
| Clavicle, - - - - - | 3 | 0 | 0 | 3 |
| Scapula, - - - - - | 0 | 1 | 0 | 1 |
| Condyles of humerus, - - - | 4 | 0 | 0 | 4 |
| Forearm, (both bones,) - - - | 2 | 0 | 0 | 2 |
| Ulna, - - - - - | 1 | 0 | 0 | 1 |
| Radius, - - - - - | 4 | 1 | 0 | 5 |
| Metacarpal bones, - - - - - | 0 | 1 | 0 | 1 |
| Vertebræ, - - - - - | 1 | 0 | 0 | 1 |
| Femur, - - - - - | 0 | 0 | 1‡ | 1 |
| Patella, - - - - - | 1‖ | 0 | 0 | 1 |
| Leg, (both bones,) - - - - - | 2 | 0 | 0 | 2 |
| Tibia, - - - - - | 1 | 0 | 0 | 1 |
| Fibula, - - - - - | 1 | 0 | 0 | 1 |
| Os Calcis, - - - - - | 1 | 0 | 0 | 1 |
| Fractures, compound, 9, viz: | | | | |
| Hand, - - - - - | 1 | 0† | 0 | 1 |
| Fingers, - - - - - | 5 | 0† | 0 | 5 |
| Leg, - - - - - - | 0 | 1** | 0 | 1 |
| Foot, - - - - - - | 0 | 1 | 0 | 1 |
| Toes, - - - - - - | 0 | 1 | 0 | 1 |
| Foreign body in nares, - - - | 1 | 0 | 0 | 1 |
| Gonorrhœa, - - - - - | 3 | 0 | 0 | 3 |
| Hydrocele, - - - - - | 0 | 2†† | 0 | 2 |
| Inflamed hand, - - - - - | 1 | 0 | 0 | 1 |
| Inverted nail, - - - - - | 0 | 1 | 0 | 1 |
| Iritis, (simple,) - - - - - | 0 | 1 | 0 | 1 |

* Exhausted by profuse discharge and diarrhœa.
† By amputation.
‡ Complicated with fracture of leg and ribs, and with wound of liver.
‖ Bony union ; two months under treatment.
** Amputation of leg was performed ; patient removed two days after by a friend.
†† Complicated with disease of testicle.

| | Cured. | Relieved. | Died. | Total. |
|---|---|---|---|---|
| Iritis (syphilitic,) - - - - - | 1 | 0 | 0 | 1 |
| Mania a potu, - - - - - | 0 | 0 | 2* | 2 |
| Onychia, - - - - - | 1 | 2 | 0 | 3 |
| Rupture of bladder, - - - - - | 0 | 0 | 1 | 1 |
| Swelled testicle, - - - - - | 1 | 0 | 0 | 1 |
| Syphilis, (primary,) - - - - - | 3 | 1 | 0 | 4 |
| " (secondary,) - - - - - | 5 | 2 | 0 | 7 |
| " (tertiary,) - - - - - | 0 | 1 | 0 | 1 |
| Tinea capitis, - - - - - | 1 | 1 | 0 | 2 |
| Tumor of breast, - - - - - | 0 | 1 | 0 | 1 |
| Ulcers, - - - - - | 6 | 0 | 0 | 6 |
| Wounds, incised, - - - - - | 9 | 4 | 0 | 13 |
| " " of throat, - - - | 1 | 0 | 0 | 1 |
| " lacerated, - - - - - | 9 | 3 | 0 | 12 |
| " gunshot, - - - - - | 0 | 1 | 0 | 1 |
| " penetrating, - - - - - | 1 | 0 | 0 | 1 |
| " " of abdomen, - - | 1 | 0 | 0 | 1 |
| " poisoned, - - - - - | 1 | 0 | 0 | 1 |
| | 91 | 33 | 7 | 131 |

One of the cases of dislocation of the hip, was of nine days standing, occurring in a stout Scotchman, aged 63.

The dislocation was upwards and backwards on the dorsum of the ilium, and required one hour to effect its reduction.

Patient was discharged well, fourteen days after.

The other case was that of a boy, aged 13 years. He was brought into the Hospital within a few hours after the occurrence of the accident, and the reduction was effected without difficulty.

Discharged, ten days after, well.

*Fracture of the spinous processes of the first two lumbar vertebræ. Recovery.*

A young woman aged 18, whilst engaged in washing a window in the second story of a house lost her balance and was precipitated on to the pavement.

The accident occurred on the 25th of June, in the neighbourhood of the Hospital, and I saw her within a half hour afterwards. She was suffering excruciating pain in the lumbar region. On examination, the spinous processes of the first and second lumbar vertebræ were found projecting directly backward, and

* Both hard drinkers; admitted for accidents, one with comminuted fracture of the femur, the other with severe gunshot wounds of the face; delirium manifested itself on the second day after admission, and death occurred within ten days.

by pressure on them *crepitus* could be distinctly perceived. There was no paralysis of the lower extremities, nor did any symptoms supervene afterwards to lead to the suspicion that the fracture had extended any further than through the spinous processes—there were no other injuries. Perfect rest on the back was enjoined, and the free use of morphia resorted to for the relief of the pain. Pain subsided toward the close of the following day. At the end of four weeks from the time of the accident, the union of the fragments was very firm—a considerable amount of callus had been thrown out—and the two spinous processes projected a little beyond the natural curve of the vertebral column.

Patient was discharged well, August 1st, thirty-seven days after the accident.

Her recovery was complete.

*Penetrating wound of the Abdomen—protrusion of Omentum—its removal—Recovery.*

The case of penetrating wound of the abdomen, was attended with protrusion of omentum—the treatment resorted to not being that in ordinary practice, and the favorable result attending it, sufficiently justify a full history of the case.

Mary Ann Lloyd, colored, aged 21, was admitted into the Hospital on Saturday, June 21st, at 11½ P. M., with a stab in the left groin, which she had received half an hour previous, from a person with whom she had some altercation in an Alderman's office. Skin was warm and moist; pulse good; complained of considerable pain. The wound was about three-fourths of an inch long, about half an inch above, and parallel to, Poupart's ligament—a line drawn from the anterior superior spinous process of the ilium to the umbilicus, would exactly bisect it. From it, protruded a piece of omentum of about the size of the palm of my hand, slightly congested, but otherwise healthy in its appearance. Careful efforts were made to return it, and were partially successful; but the restlessness of the patient was such as to force out as much as I would return. The prospect of success caused these efforts to be continued for half an hour, when it was deemed advisable to desist from them; the omentum had then become so much congested that it was not thought even safe to return it by dilating the wound. It was therefore concluded to cut it off. This was done—having previously passed a double ligature through the mass, close to the integuments, and strangulating it. The strangulating of the omentum was attended with very little pain to the patient. The wound was then closed by interrupted sutures. Within the sutures was included the portion of omentum strangulated by the double ligature. The object of this was, to prevent its return with the ligatures into the cavity of the abdomen. Over the wound was placed a piece of cotton cloth spread with cerate, and retained by one or two adhesive strips. Ordered, Liq. Morph. Sulph. ʒj. To be repeated every two hours, until sleep is induced.

*Sunday morning, the 22d.*—Required but ʒij. of the solution of morphia to induce sleep, which was tranquil through the whole night. Pulse 90, full and strong; skin, moist and warm; tongue clean: still complains of pain in the abdomen. Ordered, Hydrarg. Chlor. Mit. gr. ij. Pulv. Opii. gr. ss. To be repeated four times during the day. Fomentations with hops to the abdomen. Diet, barley water.

6½ P. M.—Has been dozing all day; does not complain of any pain; abdomen perfectly flaccid and free from tympanitis. Omitted the fomentations. Continued the calomel and opium. Pulse 88—full and strong.

*Monday, 23d.*—Slept well through the night. Purged slightly by the calomel. Pulse 95, less strong than yesterday. Tongue slightly furred—complains of slight pain in the side opposite to the wound, but of none in its neighborhood. Abdomen perfectly flaccid. Reduced the quantity of calomel—thus,

Hydrarg Chlor. mit. gr. j.
Pulv. Opii. gr. ss.

To be repeated four times during the day. Diet, same as before.

*Tuesday, 24th.*—Rested well through the night. Pulse 85, no pain or uneasiness—mouth slightly sore. Omitted the calomel. Diet, barley water.

*Wednesday, 25th.*—Still entirely free from pain. Wound has very much diminished in size—there is a slight discharge from the cut surface of the omentum. No tension of abdomen—merely a small ring around the wound, harder than natural. Applied flax-seed poultice—diet, gruel.

*Thursday, 26th.*—On visiting the ward last evening found the patient walking about with a blanket over her shoulders, assigning as her reason for doing so, that she was tired of her bed. This morning the edges of the wound show some disposition to slough, otherwise the patient is doing well. Pulse natural, no febrile excitement—no tension of abdomen—appetite good. Ordered full diet.

*July 1st.*—Has been doing well since last report. The slough has entirely separated, leaving an ulcer about two inches long and one inch broad: the ligatures of the omentum came away this morning.

*August, 2d.*—The wound has been healed for some days now, having granulated from the bottom—the cicatrix is small, firm, and linear in form. Patient discharged, well.

ADDINELL HEWSON, Resident Physician.

*Pennsylvania Hospital, August 15th,* 1851.

